# Eustachian and Tricuspid Valve Endocarditis: A Rare Consequence of the Automatic Implantable Cardioverter-Defibrillator Placement

**DOI:** 10.7759/cureus.20740

**Published:** 2021-12-27

**Authors:** Harsh Rawal, Udit Joshi, Jai Parekh

**Affiliations:** 1 Internal Medicine, Advocate Illinois Masonic Medical Center, Chicago, USA; 2 Cardiovascular Medicine, OSF Saint Francis Medical Center, Peoria, USA; 3 Cardiology, University of Iowa, Iowa city, USA

**Keywords:** intravenous drug user, tricuspid valve endocarditis, eustachian valve endocarditis, aicd, right sided infective endocarditis

## Abstract

Eustachian valve (EV) is usually a rudimentary structure in adults. It is an embryological remnant of sinus venosus that directs oxygenated blood from the inferior vena cava across the foramen ovale and into the left atrium. Intravenous drug use is most commonly associated with infective endocarditis of the right-sided heart structures. Other documented causes of such an occurrence are intracardiac devices like pacemakers and central venous catheters. Patients presenting with concerns of infection and embolic phenomenon should promptly undergo evaluation for infective endocarditis. Although an embryological remnant, the eustachian valve normally regresses after birth, except in a minority of the patients, it persists as a vestigial structure. Here we present an unusual case involving infective endocarditis of the eustachian valve and tricuspid valve both in a patient with recent automatic implantable cardioverter-defibrillator (AICD) placement and history of IV drug abuse and its systemic consequences in a patient with patent foramen ovale.

## Introduction

Eustachian valve, also known as the valve of the inferior vena cava, is an embryological remnant of sinus venosus. On echocardiography, the valve, if prominent, can divide the right atrium into two separate chambers. In adults, it is considered a non-functional rudimentary structure. Endocarditis involving the eustachian valve is rare and usually associated with IV drug use, mechanical devices like pacemakers, or central venous catheters [1]. Instances of association between eustachian valve endocarditis (EVE) with tricuspid valve endocarditis (TVE) in the past have been reported [2]. The literature involving eustachian valve endocarditis involves only limited cases, Alreja et al. [3] have discussed a case series describing two patients with EVE at two different age spectrums. Less than 30 cases have been reported in the literature involving EVE and even fewer with EVE and TVE combined. The age of the patients has ranged from 22-76 years, with a male to female ratio of 5:3 [3]. Here we present a case of EVE and TVE within four weeks of AICD placement and a history of ongoing IV drug use.

## Case presentation

A fifty-five-year-old male with a history of intravenous drug abuse and non-ischemic cardiomyopathy presented to the emergency room with thick pink discharge from the incision site of his recent AICD that was placed four weeks ago. He also complained of subjective fevers, chills, and rigors. He had recently completed a 10-day antibiotic course for a left thigh abscess. On admission, he was started on antibiotics of vancomycin and piperacillin/tazobactam and aggressive fluid resuscitation for possible sepsis. On day two, the patient started complaining of severe stabbing chest pain, which was located substernally without any radiation. On examination, he was afebrile with blood pressure (BP) of 99/64 and a heart rate (HR) of 72. His respiratory rate was 32, and oxygen saturation of 98% with bilevel positive airway pressure (BiPAP). He had thick bloody discharge from the site of the pacemaker pocket, and pulmonary examination showed bilateral coarse breath sounds. His clinical examination was otherwise unremarkable. He was then transferred to the intensive care unit for further care. His infected automatic implantable cardioverter-defibrillator (AICD) was removed. In the ICU, the patient was subsequently intubated for worsening respiratory failure. A complete blood count showed a normal white cell count; however, he had significant bandemia of 29% (Ref normal 0-10%). Basic metabolic profile was unremarkable. Lactic acid was 2.9. ECG showed normal sinus rhythm. Blood cultures grew gram-positive cocci with confirmed methicillin-resistant staphylococcus aureus (MRSA) DNA. A chest X-ray revealed concern for new infiltrate vs. possible septic emboli. CT scan of the chest demonstrated numerous cavitary lesions on bilateral lung fields superimposed on multifocal areas of consolidation and ground-glass attenuation concerning for septic emboli. A transthoracic echocardiogram was done, which showed severely reduced ejection fraction (EF) of 15-20% with severe global hypokinesis of the left ventricle. For better visualization of the valves, a transesophageal echocardiogram (TEE) was done, which did not show any vegetation initially. After lead removal, 3D TEE demonstrated improved EF to 40% with a 12 x 35 mm echodense structure at the junction of superior vena cava (SVC) and right atrial (RA) concerning for vegetation. The tricuspid valve also showed vegetation flopping inside the right atrium (Video 1).

**Video 1:** Transesophageal echocardiogram demonstrating eustachian and tricuspid valve endocarditis Transesophageal echocardiogram showing the presence of the eustachian valve, anatomically opposite to the inter-atrial septum with vegetation attached to it. The tricuspid valve demonstrates vegetation bulging into the right atrium with the closure of the valve.

**Figure 1:**
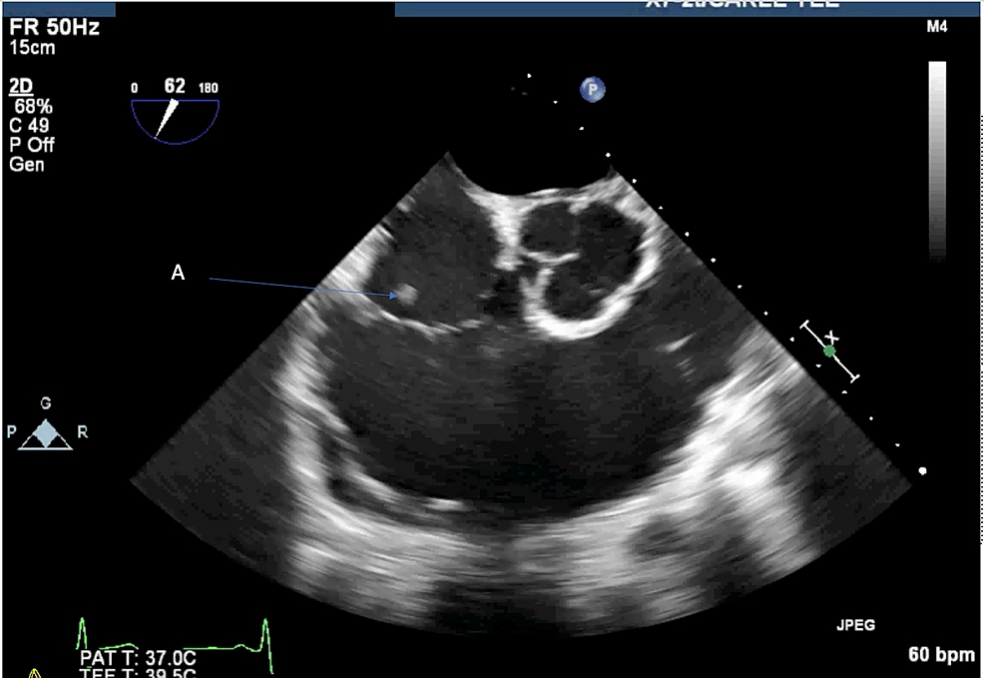
Transesophageal echocardiogram showing tricuspid valve endocarditis Transesophageal echocardiogram showing tricuspid valve endocarditis with moving into the right atrium with valve closure [A].

Another highly mobile echodensity was noted on the tricuspid valve, a 5 x 9 mm vegetation bulging into the right atrium (Figure 1).

Color Doppler demonstrated patent foramen ovale. Given the possibility of infected pacemaker site with septic emboli to the lungs it was decided to remove the pacemaker along with the leads. Subsequently, patient was continued on vancomycin for MRSA as blood cultures remained positive. Given his persistent bacteremia, ceftaroline and rifampin were added to vancomycin. Blood cultures finally were negative on day 9. He was continued on vancomycin, ceftaroline and rifampin for a total of 4 weeks. He gradually improved and was discharged to a rehabilitation unit after his prolonged stay in the ICU.

## Discussion

During the development of a normal fetal cardiovascular system, the eustachian valve (EV) is situated between the inferior vena cava and the right atrium. It directs blood flow in the fetal circulation from the vena cava through the foramen ovale into the left atrium. After birth, the valve usually regresses and becomes non-functional. The persistence of EV in adulthood is not uncommon and is usually encountered as an echocardiographic curiosity. Endocarditis associated with the EV is commonly associated with intravenous drug usage, prolonged central venous catheters, and recent abscess; however, there has been only one case reported of AICD associated EVE [4]. Involvement of other valves in addition to EV was also reported; six cases had concomitant TVE, two cases had additional mitral valve (MV) vegetations, one patient had pulmonary valve (PV) affected, and one patient had PV, tricuspid valve (TV), and MV affected [3].

Previously, cases have described methicillin-sensitive Staphylococcus aureus (MSSA) endocarditis in a patient with EV and recent AICD placement [4]. The incidence of EVE is not well reported, as it is usually underdiagnosed. In previous studies, it has been shown that tricuspid valve is the most common valve associated with endocarditis in IV drug abusers followed by mitral valve. Staphylococcus aureus is the most common organism associated with intravenous drug abuse (IVDA) and indwelling catheters. Other reported organisms that have been reported include *Staphylococcus hominis*, *Enterococcus cloacae*, *Escherichia coli*, *Proteus vulgaris*, streptococcus viridans, *Klebsiella pneumonia,* and *Actinomyces israelii.*

Transthoracic echocardiogram (TTE) is the initial diagnostic modality but has a low yield for identifying cardiac structures such as the EV due to its anatomical location [3,5]. At present, TEE is regarded as the diagnostic modality with the highest specificity, as it allows for better visualization of this embryonic remnant. As noted by Alreja et al., only a handful of cases had TTE successfully diagnosed the EVE, compared to the majority of them, which were only seen on TEE. TEE has superiority over TTE in separating pathological masses from normal structures [3].

AICD-associated EV endocarditis may become more common as there has been an increase in the number of AICDs placed over the last few years [6]. Studies show that skin flora is the most common pathogen associated with AICD-related endocarditis. Of these pathogens, methicillin-resistant Staphylococcus aureus (MRSA)-related endocarditis is relatively more common [4]. The infections have shown to not occur immediately, but rather occur after several weeks to months, as was the case in our patient. Early risk factors for AICD-associated endocarditis include fever before device implantation, repeat replacement of the device or revision, in patients without postoperative antibiotic prophylaxis.

The reviewed cases all showed improvement in functional status following appropriate antibiotic treatment with resolution of the vegetations. Vancomycin is the drug of choice for MRSA; however, given the rise in vancomycin resistance other agents such as ceftaroline, linezolid and tigecycline have been used individually or in combination for better outcomes. In patients who did not respond to medical therapy or had evidence of septic emboli and/or congestive heart failure, surgical intervention was preferred.

In summary, the patient did not improve on vancomycin alone despite appropriate microbiological sensitivities, thus warranting addition of ceftaroline as they both have synergistic activity and also since ceftaroline potentiates the activity of vancomycin. Our patient showed remission of the infectious process after completing four-week treatment and did not require surgical intervention.

## Conclusions

In conclusion, endocarditis of the EV is an under-diagnosed entity, and it may be beneficial to look for this rudimentary structure during echocardiography procedures, especially TEE, for the presence of any vegetations. With increasing AICD placement in the United States and increased prevalence of IV drug use, the possibility of detecting EV endocarditis following intra-cardiac device use may rise. 
